# Healthcare professionals show high AI enthusiasm but limited knowledge: A cross-sectional study

**DOI:** 10.1371/journal.pdig.0001433

**Published:** 2026-06-05

**Authors:** Nada Abedin, Peter Hahn, Stefan Zeuzem, Georg Dultz

**Affiliations:** 1 Medical Clinic 1, University Hospital Frankfurt, Goethe University, Frankfurt am Main, Germany; 2 Vulpius Klinik, Bad Rappenau, Germany; Zhujiang Hospital of Southern Medical University, CHINA

## Abstract

Artificial Intelligence (AI) is rapidly transforming medical practice, with successful integration critically depending on healthcare professionals’ self-reported perceived knowledge, attitudes, and perceived barriers to adoption. Despite rapid technological advances, comprehensive assessments of healthcare professionals’ AI readiness remain limited, particularly regarding the relationship between enthusiasm and self-reported competency. We conducted a cross-sectional online survey of 148 healthcare professionals. The survey assessed demographics, AI knowledge/attitudes (12 Likert-scale items), institutional readiness, learning preferences, and perceived barriers. Inferential statistics including correlation analyses, Kruskal-Wallis H tests, and Mann-Whitney U tests were performed to examine relationships between attitudes and contextual factors. Participants (mean age 36.7 ± 8.1 years, 61.5% male, 77.7% from Germany) demonstrated a significant knowledge-enthusiasm gap. While 86.5% believed AI will transform medical practice and 76.4% expressed excitement about AI changes, only 20.3% felt well-informed about healthcare AI and 38.6% had medical AI experience. Correlation analysis revealed strong positive associations among enthusiasm measures (r = 0.63-0.88, p < 0.001) but weak correlations between knowledge and enthusiasm (r < 0.20), providing evidence consistent with the knowledge-enthusiasm gap. Institutional AI stance significantly affected individual knowledge levels (Kruskal-Wallis H(3) = 28.11, p < 0.001), but not enthusiasm. Primary barriers included knowledge deficits among leadership (62.8% institutional level), infrastructure limitations (52.0%), and system integration challenges (57.4%). Healthcare professionals, particularly in a German healthcare context, demonstrate strong enthusiasm for AI integration but face significant knowledge gaps and institutional barriers. While these findings might be most directly applicable to similar healthcare contexts, the identified knowledge-enthusiasm gap represents a critical target for educational interventions in similar well-resourced European healthcare systems. Successful AI implementation requires multi-level strategies addressing leadership education, infrastructure development, and hands-on training programs. These findings provide evidence-based guidance for healthcare institutions, educators, and policymakers developing AI adoption strategies.

## Author summary

Artificial Intelligence is increasingly being integrated into healthcare, yet it remains unclear whether healthcare professionals are adequately prepared for this transition. We surveyed 148 healthcare professionals, primarily in Germany, and found a striking discrepancy: while the vast majority expressed enthusiasm about AI’s potential to transform medical practice (86.5%) and were excited about its implementation (76.4%), far fewer felt well informed about AI in general (31.3%) or in healthcare specifically (20.3%). This “knowledge-enthusiasm gap” was consistent across age groups and persisted regardless of geographic context. Another finding was that institutional support plays a critical role. Professionals at institutions actively engaging with AI reported significantly higher perceived knowledge, yet enthusiasm remained uniformly high even without institutional backing. The most frequently cited barriers were knowledge deficits among leadership, regulatory uncertainty, and integration challenges with existing systems. These findings suggest that healthcare systems need to invest in structured AI literacy programs, hands-on training opportunities, and governance frameworks to ensure that professional enthusiasm translates into safe and effective AI adoption.

## Introduction

Artificial intelligence is rapidly changing medicine and being called the steam engine of a medical revolution [1]. It is one of the most transformative developments in modern medicine [2,3]. Be it imaging analysis, histological assessments, clinical decision support or the support of drug discovery, AI is finding its way into all these fields [2–5]. However, the successful implementation of AI technologies relies critically on healthcare professionals possessing adequate AI literacy to understand capabilities, recognize limitations, and maintain appropriate clinical oversight.

Adoption success depends on end-user acceptance, organizational readiness and systematic approaches to change management. Although AI is advancing significantly, adoption rates vary significantly and remain drastically low across healthcare settings, suggesting that technology alone is not sufficient for widespread implementation [4,6].

Despite the rapid proliferation of healthcare AI technologies, systematic assessment of professional readiness remains limited. Existing studies have focused primarily on general attitudes toward AI adoption, specific applications or specialties without comprehensively examining the relationship between professional enthusiasm and actual competency, thus limiting generalizability across healthcare settings [4,7–9]. This lack in understanding is particularly concerning given the high-stakes nature of healthcare decisions and the potential for AI tools to be misused, over-relied upon, or inappropriately applied by inadequately trained professionals. Importantly, much of the existing literature relies on self-reported perceptions of AI knowledge rather than objective assessments, and research on the Dunning-Kruger effect suggests that self-assessed competency may diverge substantially from actual ability [10].

In other technology adoption contexts, a mismatch between knowledge, excitement and application has been shown where user excitement for new technologies outpaces understanding of proper implementation and limitations.

The adoption of AI in healthcare can be understood through established technology acceptance frameworks. The Unified Theory of Acceptance and Use of Technology (UTAUT) identifies performance expectancy, effort expectancy, social influence, and facilitating conditions as key determinants of technology adoption [11]. In the context of healthcare AI, professional enthusiasm may reflect high performance expectancy, i.e., the belief that AI will be beneficial, while limited self-reported knowledge may indicate low effort expectancy, a mismatch that could impede effective adoption. Similarly, Rogers’ Diffusion of Innovations framework suggests that the adoption of new technologies proceeds through stages from awareness to implementation, with the gap between awareness and persuasion representing a critical transition that determines whether adoption ultimately succeeds. While this study was not designed to formally test these models, the multi-dimensional assessment of knowledge, attitudes, institutional readiness, and barriers can be interpreted through these theoretical lenses.

The global nature of healthcare AI development and deployment requires understanding of professional readiness across diverse healthcare systems, cultural contexts, and regulatory environments, though most existing studies, including the present one, are limited to specific national contexts. Variations in educational backgrounds, institutional support, and technological infrastructure may create uneven patterns of AI readiness that influence both adoption success and patient safety outcomes [12,13].

This study addresses critical knowledge gaps in understanding healthcare professional AI readiness, with data drawn primarily from a German healthcare context, supplemented by respondents from 17 additional countries. Through comprehensive assessment across multiple domains the study aims to characterize the current state of professional readiness and provide evidence-based guidance for targeted AI education and implementation strategies.

## Methods

### Study design and participants

We conducted a cross-sectional online survey of healthcare professionals globally. Participants were recruited through professional medical networks, healthcare organizations, medical associations, and targeted social media outreach between September 2024 and January 2025. Inclusion criteria required current employment in healthcare settings in a clinical, research, or administrative capacity, or current enrollment in medical training programs. This included physicians, nurses, medical residents, medical students, researchers and healthcare administrators directly involved in patient care delivery or healthcare operations. Participants provided voluntary consent for participation and publication of anonymized results. Individual informed consent was obtained electronically via an explicit consent checkbox prior to accessing survey items. Of 214 individuals who accessed the survey, 148 (69.2%) provided complete data meeting all inclusion criteria.

### Ethics approval statement

Ethical approval was waived by the ethics committee of University Hospital Frankfurt as the study did not qualify as human biomedical research (decision 2025–2617). This study involved a voluntary and anonymous survey conducted among physicians. According to applicable national and institutional guidelines, ethical approval was not required for this type of non-interventional, survey-based research that does not involve patients or identifiable personal data. No incentives were provided, and all data were analyzed in aggregate form to ensure confidentiality.

### Survey instrument development

The survey instrument was developed through an iterative process involving input from healthcare professionals, AI researchers, and medical education experts. Expert review assessed content relevance, clarity of item wording, and comprehensiveness of response options. Pilot testing evaluated validity and completion feasibility. Refinements were made based on expert and pilot participant feedback to improve clarity and clinical relevance. The final survey consisted of 20 primary questions organized into six expert-reviewed domains addressing different aspects of AI readiness and adoption (S1 File). While formal psychometric validation (e.g., factor analysis, test-retest reliability) was not conducted prior to deployment, post-hoc internal consistency analysis was performed and is reported in the Results.

The demographics and professional characteristics section captured age, gender, highest education level, primary country of practice, institution type, years of medical experience, current patient treatment involvement, and primary medical specialty.

AI knowledge and experience assessment included both objective measures of AI tool awareness and subjective self-assessment of knowledge levels. In this study, we operationalize “perceived AI knowledge” as healthcare professionals’ self-assessed familiarity with AI concepts, measured through Likert-scale items assessing agreement with statements such as “I feel well informed about AI in general” and “I feel well informed about AI in healthcare”. It is important to note that this captures self-perceived familiarity rather than objectively tested technical competence, applied clinical understanding or ethical awareness.

Participants identified specific AI tools crucial to their daily practice and rated their agreement with 12 statements about AI knowledge, experience, and competency using five-point Likert scales ranging from strongly disagree (1) to strongly agree (5).

Institutional AI readiness was evaluated through questions about organizational stance toward AI adoption, availability of support and training programs, and institutional barriers to implementation.

Learning preferences and educational needs were addressed through questions about current information sources for AI learning and preferred modalities for future education. Barriers to AI implementation were assessed at both general healthcare system levels and specific institutional levels, allowing comparison between perceived systemic challenges and locally experienced obstacles.

The AI applications and future engagement section focused on perceived benefits of AI across 16 specific clinical and administrative applications using five-point rating scales (1 = not useful, 5 = very useful), interest in various forms of AI-related professional development, and willingness to participate in AI research and implementation activities.

### Data collection and management

Data were collected using a secure online survey platform “SurveyPlanet” with appropriate privacy protections and data encryption. The survey was available in English. Participants completed the survey voluntarily with no compensation provided, and all responses were anonymized prior to analysis. Data quality checks included identification of incomplete responses, assessment of response consistency, and detection of potential duplicate submissions.

### Statistical analysis

Descriptive statistics were calculated for all variables, with continuous variables presented as means ± standard deviations and categorical variables as frequencies and percentages.

Inferential statistics were applied to test key hypotheses. Correlation analyses employed Pearson correlation coefficients to assess relationships between attitude items, with confidence intervals calculated using Fisher’s z-transformation. As Likert-scale data are ordinal in nature, Spearman rank-order correlations were computed as a sensitivity analysis alongside Pearson correlations. Results were highly consistent between the two methods, and Pearson values are reported as the primary analysis given the 5-point scale and established practice of treating such data as approximately interval, with Spearman values provided for transparency. The correlation analyses were exploratory in nature; no correction for multiple comparisons was applied, consistent with the descriptive and hypothesis-generating aims of this study. Group comparisons utilized Kruskal-Wallis H tests to compare attitudes across institutional readiness categories (4 groups: already using AI, ready to engage, interested but reluctant, not part of conversation). Mann-Whitney U tests compared attitudes between German respondents and those from other countries. Age-related patterns were examined by comparing application ratings across three age groups (≤35, 36-50, > 50 years). Internal consistency of the Likert-scale items was assessed using Cronbach’s alpha.

Statistical significance was set at α = 0.05 (two-tailed). Effect sizes were calculated and reported as correlation coefficients (r) for both correlation analyses and Mann-Whitney U tests, and as eta-squared (η²) for Kruskal-Wallis tests (small: 0.01, medium: 0.06, large: 0.14). All tests included 95% confidence intervals where applicable.

All analyses were conducted using Python (version 3.14.0) with pandas (version 3.0.0) for data manipulation, SciPy (version 1.17.0) for statistical tests, NumPy (version 2.4.2) for numerical computations, and matplotlib (version 3.10.8) and seaborn (version 0.13.2) for visualizations.

## Results

### Participant characteristics

A total of 148 healthcare professionals from 18 countries completed the survey. Participant characteristics are presented in Table 1. The mean age was 36.7 ± 8.1 years (range 21-67). Age distribution revealed a strong concentration in early-to-mid career professionals, with 77.7% of participants between ages 21-40 years (58.1% aged 31-40, 19.6% aged 21-30). Male participants comprised 61.5% of the sample.

**Table 1:** Participant Demographics and Professional Characteristics; Multiple responses allowed for education, here participants were classified by their highest degree according to the hierarchy: Professor’s degree > MD-PhD > PhD/Doctorate > MD > Master’s > Bachelor’s > Medical Student. PhD category combines Doctorate and PhD degrees (equivalent qualifications).

Characteristic	n (%) or Mean ± SD
**Total Participants**	148
**Age Distribution**	
Mean age (years)	36.7 ± 8.1
Age range (years)	21-67
Valid age responses	148 (100.0%)
**Age Categories**	
21-30 years	29 (19.6%)
31-40 years	86 (58.1%)
41-50 years	21 (14.2%)
51-60 years	10 (6.8%)
61 + years	2 (1.4%)
**Gender Distribution**	
Male	91 (61.5%)
Female	57 (38.5%)
**Education Level** (n = 148, highest degree)	
Professor’s degree	9 (6.1%)
MD-PhD	12 (8.1%)
PhD	51 (34.5%)
MD	56 (37.8%)
Master’s degree	11 (7.4%)
Bachelor’s degree	5 (3.4%)
Medical Student	1 (0.7%)
High school graduate	1 (0.7%)
Unknown	2 (1.4%)
**Geographic Distribution** (n = 148)	
Germany	115 (77.7%)
United States	8 (5.4%)
Canada	3 (2.0%)
Italy	3 (2.0%)
Saudi Arabia	3 (2.0%)
Austria	2 (1.4%)
Pakistan	2 (1.4%)
Spain	2 (1.4%)
**Other countries (10 countries)**	10 (6.8%)
**Total Countries Represented**	18

Educational credentials were high. Participants were classified by their highest degree: Professor’s degree (6.1%), MD-PhD (8.1%), PhD (34.5%, combining Doctorate and PhD degrees), MD (37.8%), Master’s degree (7.4%), Bachelor’s degree (3.4%), and Medical Student (0.7%).

Regarding geographic distribution, 77.7% of participants were from Germany (115 of 148), while the remaining 22.3% represented 17 additional countries across multiple continents, including developed healthcare systems (United States, Italy, Canada, Austria) and emerging healthcare markets (Pakistan, Saudi Arabia, India).

### AI Knowledge, attitudes, and experience

**Fig 1:**
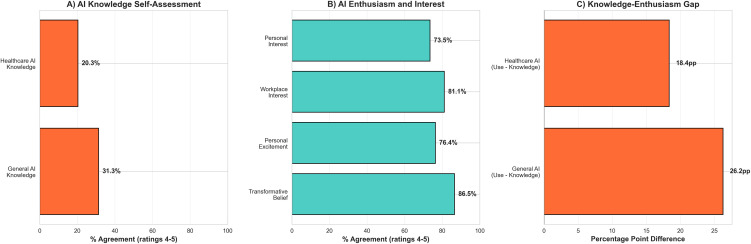
Knowledge-enthusiasm gap; (A) AI Knowledge Self-Assessment, agreement shown as percentages. **(B)** AI Enthusiasm and Interest about AI changes, agreement shown as percentages. **(C)** AI-Knowledge and Enthusiasm. Numbers given in percentage-point-differences.

Assessment of AI attitudes revealed high enthusiasm alongside limited knowledge (Table 2, Fig 1). Regarding transformative potential, 86.5% of respondents agreed that AI will change medical practice and 76.4% reported excitement about AI changes in healthcare. Interest levels remained high, with 81.1% expressing interest in using AI at workplace and 73.5% reporting personal interest in learning about AI.

**Table 2:** AI Knowledge and Attitudes Assessment, n = 148 respondents, percentages = (n/148)×100; *high agreement answers were answers rated 4 and 5.

Domain	Measure	Mean ± SD	High Agreement (4–5)*
**AI Knowledge Self-Assessment**			
General AI Knowledge	I feel well informed about AI in general	3.0 ± 1.1	31.3%
Healthcare AI Knowledge	I feel well informed about AI in healthcare	2.5 ± 1.2	20.3%
**AI Experience**			
General AI Use	I have tried using AI applications (e.g., Large Language Models (LLMs) before	3.4 ± 1.5	57.5%
Medical AI Use	I have tried using AI applications in my medical practice before	2.8 ± 1.5	38.6%
**AI Enthusiasm and Beliefs**			
Transformative Belief	I believe AI will change medical practice	4.3 ± 0.9	86.5%
Personal Excitement	I am excited about the changes AI will bring to the medical field	4.1 ± 1.0	76.4%
Workplace Interest	I would love to try new AI applications at my workplace	4.2 ± 1.0	81.1%
Personal Interest	I would love to try new AI applications in my own daily life	4.0 ± 1.1	73.5%
**AI Benefits and Support**			
Clinical Support	I believe AI will help support doctors in patient care	4.2 ± 0.9	85.1%
Patient Benefits	I believe patient care could significantly benefit from AI	4.2 ± 0.9	84.5%
Professional Engagement	Medical professionals should be engaged in AI development	4.3 ± 1.0	82.3%
**AI Concerns**			
Safety Concerns	I am concerned that patient wellbeing/safety might be neglected	2.8 ± 1.2	30.4%

In contrast, knowledge levels were substantially lower. Only 31.3% felt well informed about AI in general and 20.3% felt well informed about AI in healthcare (Fig 1A). Prior experience using AI in medical contexts was reported by 38.6%. Professional engagement attitudes were strong, with 82.3% agreeing that medical professionals should be involved in AI development. When asked whether AI can support physicians in clinical care, 85.1% agreed, while 30.4% expressed concern that patient safety might be affected during AI implementation (Fig 1B).

Comparison of knowledge and enthusiasm scores revealed substantial gaps of 45.1 percentage points (general AI: 31.3% informed vs. 76.4% excited) and 56.1 percentage points (healthcare AI: 20.3% informed vs. 76.4% excited), demonstrating a pronounced knowledge-enthusiasm gap (Fig 1C).

### Internal consistency

Cronbach’s alpha for the 12 Likert-scale attitude items (Q11) was 0.822, indicating good overall internal consistency. Sub-scale analysis revealed excellent reliability for the enthusiasm sub-scale (6 items; α = 0.935) and the knowledge sub-scale (2 items; α = 0.886).

### Institutional AI readiness and support

Assessment of institutional AI readiness revealed significant variation in organizational approaches to AI adoption (Table 3). Among respondents, 25.7% indicated that their institution is actively using or encouraging AI, while 20.9% reported institutional readiness to engage with AI technologies. However, 25.7% described their institutions as interested but very reluctant, and 34.5% reported that AI is not part of organizational conversations at all.

**Table 3:** Institutional AI Stance and Organizational Readiness.

Institutional Stance	n
Already using AI/ encouraging the use of AI	38
Ready to engage	31
Interested, but very reluctant	38
AI is not part of the conversation	51
Others (data security, no policy, discouraged)	6

Regarding funding and support, institutional financial support for AI training or implementation was available to only 16.9% of participants. In contrast, 45.3% of respondents indicated willingness to invest personal time or resources in AI education (Table 4), highlighting a disconnect between individual motivation and organizational support.

**Table 4:** Professional Development and Engagement Interests; *high agreement answers were answers rated 4 and 5.

Engagement Area	Mean ± SD	High Interest (4–5)*
**Learning and Education**		
I would like to learn more about AI	4.4 ± 1.0	85.8%
I am interested in learning about applications in my current practice	4.3 ± 1.0	83.8%
I am interested in staying up to date on AI in healthcare topics	4.2 ± 1.0	78.4%
I am interested in attending workshops on AI in healthcare	3.8 ± 1.3	64.2%
**Research and Development**		
I am interested in performing research on AI and healthcare	3.7 ± 1.3	58.8%
I am interested in participating in AI studies	3.6 ± 1.3	57.4%
I am interested in performing trials of AI applications	3.8 ± 1.2	70.3%
**Implementation and Innovation**		
I am interested in implementing/piloting new AI applications in daily practice	3.9 ± 1.2	70.3%
I am interested in career options that connect AI to healthcare	3.3 ± 1.5	46.6%
I am interested in being connected to AI startups	3.3 ± 1.3	45.9%
I am interested in being connected to AI engineers	3.3 ± 1.4	50.7%
**Innovation and Ideas**		
I have an idea of possible AI applications to share with collaborators	2.8 ± 1.5	36.5%
**Resource Commitment**		
My institution provides funding support to pursue AI activities	2.2 ± 1.3	16.9%
I am willing to invest personal resources to enhance my AI skills	3.2 ± 1.3	45.3%

### Professional development interests and educational infrastructure assessment

Healthcare professionals demonstrated strong interest in AI-related professional development, as detailed in Table 4. 85.8% want to learn more about AI, and 83.8% were specifically interested in applications relevant to their current practice. Interest in staying current with AI developments was reported by 78.4%, and 64.2% expressed interest in attending workshops. Research participation interest was reported by 58.8% of participants and implementation activities by 70.3%.

### Barriers to AI implementation

Barriers analysis was performed at both general healthcare system and institutional levels (Table 5). The data revealed an ecosystem with challenges extending beyond simple knowledge deficits to encompass systematic organizational, technical, regulatory, and financial issues.

**Table 5:** Comprehensive Barriers to AI Implementation.

Barrier Category	General Healthcare System	Institutional Barriers
	n	n
**Knowledge-Related Barriers**		
Lack of knowledge (administration/deciders)	107	93
Lack of knowledge (providers)	75	69
**Total Knowledge Barriers**	179	162
**Technical and Infrastructure**		
Integration with existing systems	95	85
Lack of infrastructure	79	77
**Regulatory and Compliance**		
Regulatory issues	95	74
Privacy concerns	71	70
**Financial**		
Cost/budget constraints	64	80
**Organizational Resistance**		
Resistance from administration/deciders	56	42
Resistance from providers	29	20
**Other**		
Patient acceptance	28	16
No obstacles	3	3
Other	11	3

At the general healthcare system level, the most frequently reported barriers included lack of knowledge among decision-makers (107/148, 72.3%) and providers (75/148, 50.7%), integration challenges with existing systems (95/148, 64.2%), regulatory or legal concerns (64.2%), and lack of IT infrastructure (79/148, 53.4%). Privacy concerns were reported by 71/148 (48.0%) of respondents, and cost or budget constraints by 64/148 (43.2%). Patient acceptance concerns were relatively minimal (28/148, 18.9%).

At the institutional level, similar patterns emerged. Lack of knowledge among leadership (93/148, 62.8%), and providers (69/148, 46.6%) remained the most frequent barrier followed by integration challenges (85/148, 57.4%), cost or budget constraints (80/148, 54.1%), and lack of infrastructure (77/148, 52.0%). Regulatory issues (74/148, 50.0%) and data privacy concerns (70/148, 47.3%) were also prominently reported. Notably, only 3/148 (2.0%) of participants reported no barriers to institutional AI implementation.

In response to open-ended questions, several respondents mentioned lack of time and insufficient technical support, as well as concerns about liability and unclear accountability. Additionally, concerns about algorithmic bias, data protection, and lack of standardization were mentioned.

### AI application benefits assessment

**Fig 2:**
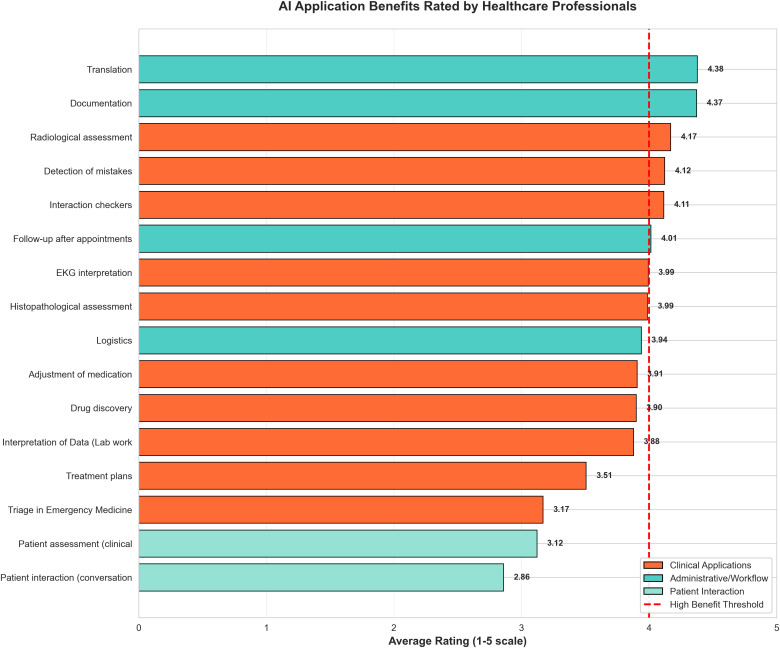
AI Application ranking by healthcare professionals; perceived benefits of various AI applications in healthcare, rated on a 1-5 scale. Applications are color-coded by category: Clinical Applications (orange), Administrative/Workflow (blue), and Patient Interaction (green). A red dashed line indicates the “High Benefit Threshold” at 4.0.

Perceived usefulness of AI applications was rated on a five-point Likert scale from 1 (not useful) to 5 (very useful), as shown in Table 6 and Fig 2. The highest-rated applications were translation services (mean 4.38 ± SD), followed closely by documentation support (4.37), radiological assessment (4.17), detection of mistakes (4.12), and medication interaction checkers (4.11).

**Table 6:** AI Application Benefits Assessment; Ratings on a 5-point Likert scale (1 = not useful, 5 = very useful). Median and interquartile range (IQR) reported alongside mean scores given the ordinal nature of the data.; *Average calculated as Score/Response Rate.

AI Application	Score	Average*	Median	IQR
**Highest Rated Applications**				
Translation	648	4.38	5.0	4-5
Documentation	647	4.37	5.0	4-5
Radiological assessment	617	4.17	4.0	4-5
Detection of mistakes	610	4.12	4.0	4-5
Interaction checkers	609	4.11	5.0	3-5
**Moderately Rated Applications**				
Histopathological assessment	590	3.99	4.0	4-5
ECG interpretation	591	3.99	4.0	3-5
Logistics	583	3.94	4.0	3-5
Drug discovery	577	3.90	4.0	3-5
Follow-up after appointments	582	4.01	4.0	3-5
**Moderately-Lower Rated Applications**				
Data interpretation (Lab work, patient history)	574	3.88	4.0	3-5
Adjustment of medication	578	3.91	4.0	3-5
Treatment plans	519	3.51	4.0	3-4
Triage in Emergency Medicine	469	3.17	3.0	2-4
**Lower Rated Applications**				
Patient assessment (clinical)	462	3.12	3.0	2-4
Patient interaction (conversation)	423	2.86	3.0	2-4

Moderate ratings were assigned to follow-up after appointments (4.01), ECG interpretation (3.99), histopathological assessment (3.99), logistics (3.94), adjustment of medication (3.91), drug discovery (3.90), and interpretation of laboratory data (3.88). Applications with lower mean ratings included treatment plans (3.51), triage in emergency medicine (3.17), patient assessment (3.12), and patient interaction conversation (2.86).

### Currently Used AI Applications

In response to an open-ended question about prior experience and use of AI in clinical context, 18.2% (27/148) of respondents indicated that they had already used AI applications in their clinical practice. The most frequently mentioned application was *ChatGPT* (n = 12), reported for tasks such as generating summaries or exploring medical topics. Other cited applications included speech recognition/scribing tools such as *Dragon Medical* or *MedSpeech*, Radiology associated AI tools and Drug-interactions checkers.

### Correlation Analysis of AI attitudes

**Fig 3:**
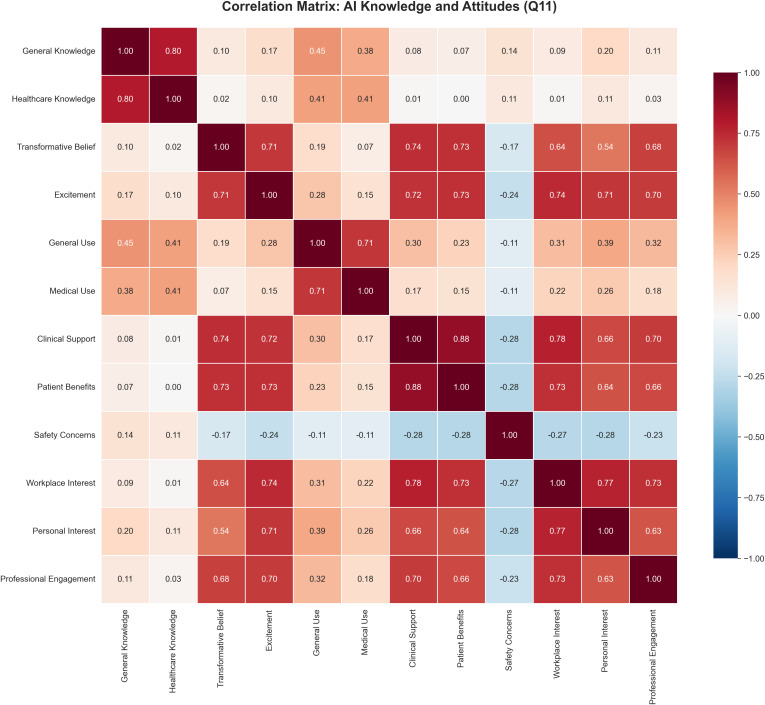
Correlation matrix of AI knowledge and attitude items. Heatmap showing Pearson correlation coefficients (r) between 12 AI attitude items. Blue shades indicate positive correlations; red shades indicate negative correlations. Darker colors represent stronger correlations. The weak correlations between knowledge items (top rows) and enthusiasm measures (r < 0.20) provide statistical evidence for the knowledge-enthusiasm gap, while strong positive correlations (r > 0.70, dark blue) indicate attitude clusters.

Correlation analysis revealed distinct attitude clusters, providing evidence consistent with the knowledge-enthusiasm gap (Table 7, Fig 3). Knowledge items showed strong positive correlations with each other (general AI knowledge ↔ healthcare AI knowledge: r = 0.796, 95% CI [0.73, 0.85], p < 0.001) but weak correlations with enthusiasm measures (general knowledge ↔ excitement: Pearson r = 0.174, p = 0.038; Spearman ρ = 0.231, p = 0.005; healthcare knowledge ↔ excitement: Pearson r = 0.101, p = 0.231; Spearman ρ = 0.178, p = 0.033). This weak relationship between knowledge and enthusiasm supports the descriptive finding of a knowledge-enthusiasm gap observed in descriptive statistics.

**Table 7:** Correlation Analysis of AI Attitudes (n = 148). Pearson correlation coefficients (r) with 95% confidence intervals calculated using Fisher’s z-transformation. Strong positive correlations (r > 0.70) indicate attitudes that cluster together; negative correlations indicate opposing relationships. Note the weak correlation between knowledge and enthusiasm measures (r < 0.20, not shown), providing evidence consistent with the knowledge-enthusiasm gap.

Attitude Pair	r	95% CI	p	Interpretation
**Strongest Positive Correlations**				
Clinical Support ↔ Patient Benefits	0.875	[0.83, 0.91]	<0.001	Very strong positive
General Knowledge ↔ Healthcare Knowledge	0.796	[0.73, 0.85]	<0.001	Very strong positive
Clinical Support ↔ Workplace Interest	0.781	[0.71, 0.84]	<0.001	Very strong positive
Workplace Interest ↔ Personal Interest	0.767	[0.69, 0.83]	<0.001	Very strong positive
Excitement ↔ Workplace Interest	0.745	[0.66, 0.81]	<0.001	Very strong positive
Transformative Belief ↔ Clinical Support	0.736	[0.65, 0.80]	<0.001	Very strong positive
Patient Benefits ↔ Workplace Interest	0.734	[0.65, 0.80]	<0.001	Very strong positive
Workplace Interest ↔ Professional Engagement	0.732	[0.65, 0.80]	<0.001	Very strong positive
Excitement ↔ Patient Benefits	0.728	[0.64, 0.80]	<0.001	Very strong positive
Transformative Belief ↔ Patient Benefits	0.727	[0.64, 0.79]	<0.001	Very strong positive
**Strongest Negative Correlations**				
Safety Concerns ↔ Personal Interest	-0.284	[-0.43, -0.13]	<0.001	Moderate negative
Patient Benefits ↔ Safety Concerns	-0.284	[-0.43, -0.13]	<0.001	Moderate negative
Clinical Support ↔ Safety Concerns	-0.284	[-0.43, -0.13]	<0.001	Moderate negative
Safety Concerns ↔ Workplace Interest	-0.274	[-0.42, -0.12]	<0.001	Moderate negative
Excitement ↔ Safety Concerns	-0.239	[-0.39, -0.08]	0.003	Moderate negative

Clinical optimism items clustered together strongly. The strongest correlation was between beliefs about AI supporting clinical care and patient benefits (r = 0.875, 95% CI [0.83, 0.91], p < 0.001). Enthusiasm measures showed very strong positive associations with workplace interest (excitement ↔ workplace interest: r = 0.745, 95% CI [0.66, 0.81], p < 0.001).

Safety concerns showed modest negative correlations with enthusiasm measures (safety concerns ↔ excitement: r = -0.239, 95% CI [-0.39, -0.08], p = 0.003), suggesting that concerns coexist with cautious optimism rather than opposing enthusiasm entirely.

### Institutional context and individual attitudes

Institutional AI stance significantly influenced individual attitudes, particularly regarding knowledge levels (Table 8). Healthcare professionals at institutions already using AI reported higher mean healthcare AI knowledge scores compared to those where AI was not part of conversations (Kruskal-Wallis H(3) = 28.11, p < 0.001, η² = 0.19). Post-hoc analyses revealed that healthcare AI knowledge increased progressively with institutional engagement level.

**Table 8:** Institutional Stance Impact on Individual AI Attitudes (n = 148). Kruskal-Wallis H tests comparing attitudes across four institutional readiness levels. Healthcare AI knowledge and prior medical AI use showed significant differences across groups, while enthusiasm remained consistently high regardless of institutional support. Effect sizes: η² = 0.01 (small), 0.06 (medium), 0.14 (large). n.s. = not significant.

Attitude Mean ± SD)	Already Using AI (n = 38)	Ready to Engage (n = 31)	Interested but Reluctant (n = 38)	Not Part of Conversation (n = 51)	H statistic (df = 3)	p-value	Effect Size (η²)
Healthcare AI Knowledge	Higher	Moderate	Moderate	Lower	28.11	<0.001	0.19 (medium)
Excitement about AI	High	High	High	High	7.15	0.067 (n.s.)	0.05 (small)
Medical AI Use	Higher	Moderate	Moderate	Lower	21.44	<0.001	0.14 (medium)
Safety Concerns	Moderate	Moderate	Moderate	Moderate	4.87	0.182 (n.s.)	0.03 (negligible)

However, enthusiasm levels remained consistently high across all institutional categories (H(3) = 7.15, p = 0.067, η² = 0.05), suggesting that individual motivation for AI learning exists independently of organizational support. Medical AI use experience showed significant institutional variation (H(3) = 21.44, p < 0.001, η² = 0.14), with higher rates in institutions actively using AI. Safety concerns did not differ significantly across institutional groups (H(3) = 4.87, p = 0.182), indicating that concerns about AI implementation are widespread regardless of institutional context.

### Stratified analyses

To assess whether the knowledge-enthusiasm gap varied across demographic subgroups, we conducted stratified analyses by age group (≤35, 36-50, > 50 years) and geographic region (Germany vs. other countries). Healthcare AI knowledge differed significantly across age groups: 17.3% of participants aged ≤35 reported high knowledge, compared with 20.0% of those aged 36-50 and 41.7% of those aged >50 (Kruskal-Wallis H(2) = 10.27, p = 0.006). In contrast, enthusiasm levels remained consistently high and did not differ significantly by age (75.3%, 74.5%, and 91.7%, respectively; H(2) = 3.36, p = 0.187), indicating that the knowledge-enthusiasm gap was most pronounced among younger professionals.

Geographic stratification revealed that German respondents (n = 115) reported lower general AI knowledge than respondents from other countries (n = 33; 25.2% vs. 53.1% high knowledge, Mann-Whitney U, p = 0.007). However, enthusiasm levels did not differ significantly between groups (73.9% vs. 84.8%, p = 0.290). These findings suggest that while the knowledge-enthusiasm gap is present across geographic subgroups, its magnitude may vary with local training infrastructure and AI exposure. The consistency of high enthusiasm regardless of geography or age supports the interpretation that motivation for AI adoption is robust, while knowledge gaps represent the primary target for intervention.

## Discussion

This study provides comprehensive insights into healthcare professionals’ readiness for AI adoption, revealing high enthusiasm and sophisticated professional judgment alongside limited practical knowledge and inconsistent institutional support. These findings highlight a pronounced gap between individual motivation and organizational preparedness, underscoring the need for targeted educational strategies and institutional enablers to support responsible AI integration into clinical practice.

The sample characteristics merit consideration when interpreting these findings. The strong concentration of early-to-mid career professionals (77.7% ages 21-40) and highly educated participants (86.5% holding Professor, MD-PhD, PhD, or MD degrees) indicates a sample weighted toward research-oriented healthcare professionals. The predominance of German respondents (77.7%) provides in-depth insight into AI readiness within a well-resourced healthcare system with advanced technological infrastructure, robust medical education systems, and supportive regulatory environments for healthcare innovation. While this geographic concentration may limit generalizability to other healthcare contexts, the systematic challenges identified likely reflect patterns relevant to similar well-resourced systems internationally.

### Knowledge-Enthusiasm gap

The comprehensive assessment of AI knowledge and attitudes revealed a critical knowledge-enthusiasm gap that forms the central finding of this study. Most participants acknowledged the transformative potential of AI in healthcare, with 86.5% agreeing that AI will change medical practice and 76.4% expressing excitement about these developments. However, only 20.3% felt well informed about AI in healthcare and 38.6% reported prior experience using AI in clinical settings. Correlation analysis provided further evidence consistent with this gap, demonstrating strong correlations among enthusiasm measures (r = 0.63-0.88) but weak correlations between knowledge and enthusiasm items (r < 0.20).

These results align with recent studies reporting similar discrepancies between perceived importance of AI and actual preparedness. Pinto Dos Santos et al. found high awareness, but low self-rated competence in AI-concepts, particularly in non-radiology specialties [14]. Furthermore, clinicians acknowledge AI’s relevance, yet fewer than one in four feels confident using AI tools [15].

The mismatch between enthusiasm and readiness may contribute to inconsistent adoption, underutilization or inappropriate reliance on AI applications. Recent studies have raised concerns about automation bias, where clinicians defer to algorithmic outputs even in the face of conflicting clinical judgement, particularly when lacking a foundational understanding of AI’s limitations [16,17]. AI deployment without adequate user understanding can result in automation bias, overreliance, and failure to detect system-errors [13,18,19], inappropriate clinical decisions, or false confidence in automated systems [18,20,21]. Understanding the current state of AI literacy among healthcare professionals is therefore essential for developing appropriate educational interventions and safety frameworks [22]. While positive attitudes are encouraging, they must be complemented by systemic training and competency development.

### Institutional readiness and support

Our findings revealed significant institutional variation in AI readiness, with only 25.7% of participants reporting that their institution was actively using or encouraging AI. Institutional stance significantly influenced individual knowledge levels (H(3) = 28.11, p < 0.001) but not enthusiasm (H(3) = 7.15, p = 0.067), demonstrating that individual motivation exists independently of organizational support. While 45.3% of participants expressed willingness to invest personal resources in AI education, only 16.9% had access to institutional financial support for AI-related training. This disconnect between individual motivation and organizational support represents a critical barrier to AI adoption.

Earlier studies have shown similar results emphasizing that even when clinicians are eager to explore AI, uptake is often limited by data governance and integration pathways [23]. Our results suggest that these barriers are not isolated, but widespread across diverse clinical environments. Addressing this gap will require not only technological infrastructure, but also institutional policies that provide access to AI training and upskilling support, as well as opportunities to engage in pilot projects and co-development of AI solutions. Importantly, 70.3% of respondents supported clinician involvement in AI product development, indicating strong willingness to contribute to technology design if given appropriate opportunities and support.

### Applications perceived as useful

Healthcare professionals demonstrate good understanding of appropriate AI applications and professional responsibilities, with 82.3% believing medical professionals should be engaged in AI development and 85.1% recognizing AI’s potential to support clinical care [13]. However, only 20.3% feel adequately informed about healthcare AI applications, and just 38.6% have meaningful practical experience. This combination of high motivation, sophisticated judgment, and limited competency raises concerns that well-intentioned professionals may adopt AI tools without sufficient understanding of their limitations, potentially affecting patient safety and reducing the likelihood of realizing technology benefits.

Translation tools, documentation support and radiology applications were rated as the most useful tools. These priorities reflect the practical orientation towards tools that support rather than replace clinical workflows and are already commonly available. Healthcare professionals prioritize practical workflow solutions that address immediate daily challenges over more technologically sophisticated applications that may receive greater attention in research and development communities. Several large-scale surveys have reported similar preferences. In the American Medical Association’s 2022 physician digital health survey, the highest-rated AI use cases were those that reduced administrative burden, such as automated documentation and information retrieval [9,24].

In contrast to that, applications related to treatment planning, emergency triage, and patient communication received lower scores. These ratings may reflect perceived implementation challenges, higher perceived risk, and unresolved ethical concerns, such as potential depersonalization of care or liability issues. Additionally, they demonstrate appropriate professional values that prioritize human connection in healthcare delivery. AI adoption will be guided by patient-centered care principles and implementation strategies should emphasize augmentation of clinical capabilities rather than replacement of human interaction and relationship management.

### Barriers to AI implementation

Among respondents, 34.5% reported that AI was not part of organizational conversations at their institution, highlighting limited engagement at the leadership level. Barriers were reported at both general healthcare system and institutional levels, with the most common including lack of knowledge among leadership (72.3% general, 62.8% institutional), integration challenges (64.2% general, 57.4% institutional), regulatory concerns (64.2% general, 50.0% institutional), and cost constraints (43.2% general, 54.1% institutional). Notably, lack of knowledge among leadership represents a systemic issue that may cascade to undertraining, underutilization, and limited implementation of available AI tools. These results mirror findings from global policy analyses and implementation research, which consistently identify structural and governance-related factors as critical obstacles to AI implementation [25].

A review by Liu et al. identified interoperability, insufficient technical infrastructure, and uncertainty about clinical validation as the most prominent system-level barriers to AI adoption [26]. Our data confirm these findings and further add nuance through free-text responses, where participants expressed concern about unclear accountability, liability in clinical decision-making, and insufficient IT support.

These barriers take on additional significance in the European regulatory context. The EU Artificial Intelligence Act (Regulation 2024/1689), which entered into force in August 2024, classifies most healthcare AI applications as high-risk, imposing requirements for conformity assessment, human oversight, transparency, and post-market monitoring. Given that 77.7% of our sample practices in Germany, these regulatory requirements are directly applicable. The finding that 64.2% of respondents identified regulatory concerns as a general barrier and 50.0% at the institutional level suggests that healthcare professionals are already aware of the regulatory complexity surrounding AI adoption, even as specific provisions of the Act are still being phased in. Addressing the knowledge-enthusiasm gap identified in our study will therefore require not only clinical AI training but also education on the evolving regulatory landscape governing AI use in healthcare.

Beyond regulatory compliance, the integration of AI into clinical workflows introduces cybersecurity and data privacy challenges that our respondents recognized as significant barriers (48.0% at the general level, 47.3% at the institutional level). Healthcare data are particularly sensitive, and AI systems that require large datasets for training and validation may increase exposure to breaches or unauthorized access. Emerging technical approaches such as federated learning, which enables model training across institutions without centralizing patient data, and differential privacy techniques offer promising solutions to balance AI utility with data protection requirements. International frameworks including the NIST AI Risk Management Framework and the WHO guidance on ethics and governance of AI for health provide complementary standards that may help healthcare institutions develop robust governance structures for AI deployment. Future research should evaluate how awareness of these technical safeguards influences healthcare professionals’ willingness to adopt AI tools, as our findings suggest that privacy and security concerns represent modifiable barriers to implementation.

Importantly, very few respondents identified clinician resistance or cultural opposition as primary barriers. This is consistent with recent literature suggesting that frontline resistance to AI is decreasing, particularly when tools are designed with clinician involvement and tailored to clinical needs. Patient acceptance concerns were among the least frequently reported barriers (28/148, 18.9% at the general level; 16/148, 10.8% at the institutional level). The confidence that patients will accept AI-assisted care is high [9,27]. This finding suggests that professional readiness represents a more critical factor than patient education for successful AI adoption, though transparent communication about AI use remains important for maintaining trust and informed consent.

### Educational needs and engagement preferences

With over 85% of participants expressing a desire to learn more, particularly in formats relevant to their clinical specialty, the willingness to learn was confirmed. Preferred modes of engagement included workshops, participation in research projects, and involvement in pilot implementations. These findings are consistent with calls from academic and professional bodies to include AI in continuing medical education and specialty training programs [28].

Despite growing interest, AI-related education remains largely unstructured. A recent review noted that only a minority of medical schools and postgraduate programs currently offer formal AI curricula, and even fewer provide practical, case-based learning [29]. As our findings suggest, clinicians are willing to invest time and effort into AI upskilling if supported with accessible, relevant, and unbiased content.

The observed preference for applied learning experiences such as workshops, simulations, or involvement in clinical pilots should inform the development of future educational strategies. Incorporating feedback from clinicians into the design and evaluation of AI training modules may further enhance engagement and relevance.

### Use of AI tools in current clinical practice

Among respondents with prior AI experience, ChatGPT was the most frequently mentioned tool, primarily used for summarization, document drafting, and patient communication. Other tools included speech recognition software, AI-assisted radiology, and drug-interaction alerts. These use cases are consistent with other early reports of large language models and rule-based AI tools entering routine clinical workflows [30].

However, the use of general-purpose models such as ChatGPT in clinical contexts also raises concerns. Several studies have highlighted issues related to hallucinations, non-determinism, and lack of clinical validation in large language models [6,31]. While such tools may offer efficiency gains, their use must be accompanied by clear boundaries, informed oversight, and regulatory guidance to ensure safe integration into healthcare environments [32].

### Strengths and limitations

This study has several strengths. It is among the first to assess AI readiness in healthcare professionals using a multi-dimensional approach that includes attitudes, institutional readiness, perceived usefulness, barriers, and free-text insights. The comprehensive analytical approach included both descriptive and inferential statistics, providing robust evidence for the knowledge-enthusiasm gap through correlation analyses and group comparisons and the survey had a high response completion rate across all domains and included participants from 18 countries, allowing for a degree of international perspective.

However, there are several limitations that should be acknowledged. First, the sample is predominantly from Germany (77.7%), which may limit the generalizability to healthcare systems with different technological infrastructure, educational resources, or regulatory environments. While the systematic nature of challenges identified suggests broader relevance, findings should be interpreted primarily within the context of well-resourced healthcare systems. The knowledge-enthusiasm gap may be more pronounced in healthcare systems with fewer educational resources, less technological infrastructure, or more limited access to AI expertise.

Second, the sample skews toward younger professionals (77.7% under age 40) and highly educated individuals (86.5% with Professor, MD-PhD, PhD, or MD degrees). This demographic profile likely reflects a more technologically engaged population and may underrepresent barriers faced by older or less research-oriented practitioners.

Third, all knowledge and enthusiasm measures in this study are based on self-report, which may not accurately reflect objective competency levels. Research on the Dunning-Kruger effect suggests that individuals with limited knowledge in a domain tend to overestimate their competence, while those with greater expertise may underestimate it. This means that the knowledge-enthusiasm gap identified here could be either larger or smaller than objective testing would reveal. Future studies should incorporate validated objective knowledge assessments alongside self-report measures to determine the true magnitude of this gap.

Fourth, while the survey instrument demonstrated acceptable internal consistency (Cronbach’s alpha: overall α = 0.822; knowledge subscale α = 0.886; enthusiasm subscale α = 0.935), it has not undergone formal psychometric validation including factor analysis, test-retest reliability assessment, or convergent and discriminant validity testing. The knowledge and enthusiasm constructs were defined operationally based on face-valid item groupings rather than empirically derived factor structures. Results should therefore be interpreted as reflecting response patterns on these specific items rather than as validated measures of underlying latent constructs.

Fifth, the completion rate among those who began the survey was 69.2% (148 of 214 initiated responses, total number of survey views: 591), and the overall response rate from the target population cannot be determined due to the open recruitment approach through professional networks and social media. This limits the assessment of non-response bias and the representativeness of findings.

Sixth, the cross-sectional design captures attitudes at a single time point during rapid AI evolution, limiting ability to assess how perspectives change with increased exposure or training. Additionally, recruitment through professional networks and social media creates potential selection bias toward digitally engaged professionals.

Despite these limitations, the study offers a timely and detailed snapshot of current perceptions, readiness, and barriers to AI adoption among healthcare professionals and provides direction for future interventions. Future research should include longitudinal assessment of AI readiness development, objective knowledge testing, and evaluation of educational interventions designed to bridge identified gaps. Multi-country studies are needed to assess cross-cultural variations in AI readiness patterns.

## Conclusion

This study demonstrates high enthusiasm but limited readiness for AI among healthcare professionals, with barriers rooted in institutional support, education, and governance. The identified knowledge-enthusiasm gap, supported by correlation analysis showing weak associations between knowledge and enthusiasm measures (Pearson r < 0.20; Spearman ρ = 0.18-0.23), has important implications for safe AI implementation. While our study did not directly measure patient safety outcomes, the combination of high enthusiasm (76.4% excited about AI) with limited knowledge (20.3% feeling well-informed about healthcare AI) suggests potential risks if AI tools are adopted without adequate training and oversight.

Healthcare professionals show clear priorities regarding useful applications and express strong demand for training but currently lack comprehensive AI readiness programs within their educational and institutional systems. Addressing this readiness gap will require aligned efforts across education, infrastructure, policy, and leadership. As healthcare systems increasingly adopt AI technologies, clinician engagement and support must be central to ensure safe, equitable, and effective implementation.

These findings, derived primarily from a German healthcare context with a sample weighted toward early-career, research-oriented professionals, highlight challenges in healthcare AI adoption that may be relevant to similar well-resourced systems, though generalizability to other settings requires further investigation, highlight challenges in healthcare AI adoption that may be relevant to similar well-resourced systems. The future of healthcare AI integration depends not only on technological advancement but also on systematic efforts to align professional enthusiasm with adequate knowledge, institutional support, and evidence-based implementation strategies. The motivation clearly exists, the challenges are well identified, and evidence-based solutions are achievable through coordinated intervention and appropriate resource allocation.

## Supporting information

S1 File Survey questionnaire. The complete questionnaire used for data collection in this study.
(PDF)
