# Supplementary material for: Healthcare professionals show high AI enthusiasm but limited knowledge: A cross-sectional study
Source: PLOS Digit Health. 2026 Jun 5;5(6):e0001433. doi: 10.1371/journal.pdig.0001433 (PMC13240888; doi:10.1371/journal.pdig.0001433)
Supplement: S1 File — The complete questionnaire used for data collection in this study. (PDF) [file pdig.0001433.s001.pdf]

Supplementary Material: Survey Questions

Q1 What is your age? *

Choose one.

Q2 What is your gender? *

- ◯ Male
- ◯ Female
- ◯ Non-binary
- ◯ Prefer not to disclose
- ◯ Other (please specify)

Choose one.

Q3 Highest level of education: *

Please select all that apply

- ☐ High school graduate, diploma or the equivalent
- ☐ Student (please specify below)
- ☐ Medical Student
- ☐ MD
- ☐ PhD
- ☐ MD-PhD
- ☐ Bachelor's degree
- ☐ Master's degree
- ☐ Doctorate degree
- ☐ Professor's degree
- ☐ Other (please specify)

Choose all that apply.

Q4 What is your main country of practice? *

Choose all that apply.

Comments

Q5 In which type of institution are you currently working in the healthcare sector? *

- ☐ Academic Hospital
- ☐ Private Hospital
- ☐ Private Practice
- ☐ Research
- ☐ Startup
- ☐ Tech-Company
- ☐ Insurance company
- ☐ Pharma company
- ☐ Medical Technology
- ☐ NGO
- ☐ Government
- ☐ Consulting
- ☐ I do not currently work in healthcare
- ☐ Other (please specify):

Choose no more than 14.

Q6 Years of experience in the medical field

Choose one.

Q7 Are you currently involved in patient treatment? *

- ○ Yes
- ○ No

Choose one.

Q8 In which area of medicine do you practice? *

- [ ] Anesthesiology
- [ ] Cardiology
- [ ] Critical Care
- [ ] Dermatology
- [ ] Emergency Medicine
- [ ] Endocrinology
- [ ] ENT
- [ ] Gastroenterology
- [ ] Internal Medicine
- [ ] OB / Gyn
- [ ] Oncology
- [ ] Ophthalmology
- [ ] Orthopedics
- [ ] Pathology
- [ ] Pediatrics
- [ ] Psychiatry
- [ ] Public Health
- [ ] Radiology
- [ ] Surgery
- [ ] Urology
- [ ] Administration
- [ ] Consulting
- [ ] Technology
- [ ] Other (please specify)

Choose all that apply.

Q9 Are there specific AI tools or products you are aware of? Please mention the top five that are crucial in your daily practice.

If none, please leave open.

Q10 Please indicate how much you agree with the following statements: *

1 = strongly disagree; 5 = strongly agree Please leave open, if not applicable

	1	2	3	4	5
I feel well informed about AI in general.	○	○	○	○	○
I feel well informed about AI in healthcare.	○	○	○	○	○
I believe AI will change medical practice.	○	○	○	○	○
I am excited about the changes AI will bring to the medical field.	○	○	○	○	○
I have tried using AI applications (e.g. LLMs) before.	○	○	○	○	○
I have tried using AI applications in my medical practice before.	○	○	○	○	○
I believe AI will help support doctors in patient care.	○	○	○	○	○
I believe patient care could significantly benefit from AI.	○	○	○	○	○
I am concerned that patient wellbeing/safety might be neglected with AI implementation.	○	○	○	○	○
I would love to try new AI applications at my workplace.	○	○	○	○	○
I would love to try new AI applications in my own daily life.	○	○	○	○	○
Medical professionals should be engaged in the development of AI tools in healthcare.	○	○	○	○	○

Q11 What is your institution's / employer's stance on AI? *

- ☐ Already using AI / encouraging the use of AI
- ☐ Ready to engage
- ☐ Interested, but very reluctant
- ☐ AI is not part of the conversation
- ☐ Other (please specify):

Choose all that apply.

Q12 What are the main resources you usually use to learn more / inform yourself about AI *

- ☐ Social Media
- ☐ Online newspaper
- ☐ Printed newspaper
- ☐ Trainings (in person)
- ☐ Trainings (online)
- ☐ Podcasts
- ☐ Scientific papers
- ☐ Conferences
- ☐ Books
- ☐ Newsletters
- ☐ Youtube
- ☐ None
- ☐ Other:

Choose all that apply.

Q13 From which resources would you like to learn more about AI? *

- ☐ Scientific publications
- ☐ Online courses
- ☐ Workshops
- ☐ Conferences
- ☐ Hand-on training
- ☐ Case studies
- ☐ Webinars
- ☐ Mentorship
- ☐ Networking with AI experts
- ☐ Other (please specify)

Choose all that apply.

Q14 In your point of view, what are the biggest barriers to embracing AI in healthcare in general? *

- ☐ Lack of knowledge (administration/deciders)
- ☐ Lack of knowledge (providers)
- ☐ Cost/ budget constraints
- ☐ Regulatory issues
- ☐ Privacy concerns
- ☐ Lack of infrastructure
- ☐ Integration with existing systems
- ☐ Resistance from administration/ deciders
- ☐ Resistance from providers
- ☐ Patient acceptance
- ☐ There are no obstacles
- ☐ Other (please specify):

Choose no more than 12.

Q15 Which of these factors applies in your own institution? *

- ☐ Lack of knowledge (administration/deciders)
- ☐ Lack of knowledge (providers)
- ☐ Cost/ budget constraints
- ☐ Regulatory issues
- ☐ Privacy concerns
- ☐ Lack of infrastructure
- ☐ Integration with existing systems
- ☐ Resistance from administration/ deciders
- ☐ Resistance from providers
- ☐ Patient acceptance
- ☐ There are no obstacles
- ☐ Other (please specify):

Choose no more than 12.

Q16 Please indicate how much you agree with the following statements: *

1 = strongly disagree; 5 = strongly agree

	1	2	3	4	5
I would like to learn more about AI.	○	○	○	○	○
I am interested in learning about applications in my current practice.	○	○	○	○	○
I am interested in resources that help me stay up to date on topics related to AI in healthcare.	○	○	○	○	○
I am interested in performing research on AI and healthcare/ participating in AI studies.	○	○	○	○	○
I am interested in career options that connect AI to healthcare.	○	○	○	○	○
I am interested in attending workshops on AI in healthcare.	○	○	○	○	○
I am interested in performing trials of AI applications.	○	○	○	○	○
I am interested in implementing/piloting new AI applications in my daily practice.	○	○	○	○	○
I am interested in being connected to AI startups.	○	○	○	○	○
I am interested in being connected to AI engineers to develop my own idea.	○	○	○	○	○
I have an idea of possible AI applications that I would like to share with potential collaborators.	○	○	○	○	○

	1	2	3	4	5
My institution provides funding support to pursue some or all of the above activities.	○	○	○	○	○
I am willing to invest personal resources to enhance my AI skills/ knowledge of AI.	○	○	○	○	○

Comments

Q17 AI use cases are already being explored in various applications in healthcare. How would you rate the benefit of AI in these applications? *

1 = no relevance; 5 = very helpful

	1	2	3	4	5
Adjustment of medication	○	○	○	○	○
Detection of mistakes	○	○	○	○	○
Documentation	○	○	○	○	○
Drug discovery	○	○	○	○	○
ECG interpretation	○	○	○	○	○
Follow-up after appointments	○	○	○	○	○
Histopathological assessment	○	○	○	○	○
Interaction checkers	○	○	○	○	○
Interpretation of Data (Lab work, patient history, ...)	○	○	○	○	○
Logistics	○	○	○	○	○
Patient assessment (clinical)	○	○	○	○	○
Patient interaction (conversation)	○	○	○	○	○
Radiological assessment	○	○	○	○	○
Translation	○	○	○	○	○
Treatment plans	○	○	○	○	○
Triage in Emergency Medicine	○	○	○	○	○

Comments

Q18 How will AI change the role of healthcare professionals? *

- ○ Significantly reduce their role, not important to have a physical doctor
- ○ Somewhat reduce their role, important to have a physical doctor
- ○ No significant change
- ○ Somewhat enhance their role, slightly supporting a physical doctor
- ○ Significantly enhance their role, significantly supporting a physical doctor

Choose one.

Comments

Q19 What will be the impact of AI on patient safety? *

- ○ Significantly reduce patient safety
- ○ Somewhat reduce patient safety
- ○ No relevant changes
- ○ Somewhat increase patient safety
- ○ Significantly increase patient safety

Choose one.

Q20 Do you have any other thoughts or concerns about AI in healthcare that you would like to share?
